# Some observations on the transeurasian language family, from the perspective of the Farming/Language Dispersal Hypothesis

**DOI:** 10.1017/ehs.2020.34

**Published:** 2020-06-17

**Authors:** Peter Bellwood

**Affiliations:** School of Archaeology and Anthropology, Australian National University, Canberra, Australia

**Keywords:** Archaeolinguistics, Farming/Language Dispersal Hypothesis, Transeurasian language family, Northeast Asia, Neolithic

## Abstract

During my attendance at the ‘Transeurasian Millets and Beans, Words and Genes’ conference in Jena (January 2019), Martine Robbeets invited me to comment on the articles that are published in this Special Collection in the journal *Evolutionary Human Sciences*. My comments are focused on the seven articles that deal with the ‘Farming/Language Dispersal Hypothesis’, one of the key theoretical constructs discussed during the conference. I consider how the hypothesis might aid an understanding of the prehistory and early history of the Transeurasian language family.

**Media summary:** The dispersal of the Transeurasian languages commenced with the development of agriculture in northeastern China.

## Introduction

I should make it clear from the start that I am not a specialist in the linguistic prehistory of northeastern Asia. My professional expertise is mainly in archaeology. My perspective on the Transeurasian language family is that of an outsider in many respects, but I hope that I can therefore be objective. I am grateful for the chance to present my views, and I thank Martine Robbeets and Chuan-Chao Wang for discussing the Farming/Language Dispersal Hypothesis so positively in their introductory article to the collection.

## The Farming/Language Dispersal Hypothesis

The Farming/Language Dispersal Hypothesis suggests that early farmers, through their high birth rates and constant desire for new territory, played important roles in spreading the foundation subgroups in many major language families with deeply shared agricultural vocabulary. This hypothesis was developed mainly in the 1980s and 1990s, partly by me using Austronesian as a major example, and partly by Colin Renfrew using Indo-European (Bellwood, 1983, 1991; Renfrew, 1987; Bellwood & Renfrew, 2002). My own personal views about how the hypothesis might have worked ‘on the ground’ were set out in my books *First Farmers* (Bellwood, 2005) and *First Migrants* (Bellwood, 2013).

The hypothesis concerns those language families that have extents much greater than those of any individual societies that are known to have existed prior to AD 1500, including states and empires. For instance, the extent of the Indo-European language family, even before the colonial era, was far greater than anything created by the activities of Alexander the Great or the Roman Empire. We can state the same about the Sino-Tibetan language family, compared with the extent of the ancient Chinese state. The existences of these language families, and many others, were on a scale far beyond that achievable by any known pre-AD 1500 human society, no matter how powerful that society might have been.

The Farming/Language Dispersal Hypothesis, by definition, concerns language families that had food production vocabularies (with terms related to agriculture or pastoralism) in their proto-languages. Such agriculturalist families include Indo-European, Austronesian, Austroasiatic, Bantu (as the most widespread branch of Niger-Congo), Afro-Asiatic, Sino-Tibetan, many American families, and, of course, Transeurasian.

Two points can be made about the early expansions of these very extensive agriculturalist/pastoralist (i.e., food producer) language families:

1. In terms of their trans-continental geographical scales, the only attested explanation for such extensive families, especially when compared with historical examples of language spread (Ostler, 2005), is that they travelled originally in the mouths of migrating populations. Nicholas Ostler gives many examples of population movement as the explanation for widespread single languages in recorded history (he does not discuss full language families), but he found few, if any, *extensive* and *permanent* language movements on the scale under discussion that depended on language shift to an outsider target language by unmoving indigenous populations. The migration/settler explanation comes not just from the comparative historical record, but is supported also from the recent explosion in the study of ancient DNA. This reveals many cases where ancient (especially early agricultural) genetic population movements attained similar geographical extents to the pre-AD 1500 distributions of many major agriculturalist language families (Reich, 2018).
2. No explanation for any major language family involving ‘elite dominance’ alone, that is, transmission by a small high status minority imposing a new language on a much larger indigenous population, carries much conviction for pre-state societies (or even for state-level ones), especially if long-term establishment of society-wide vernaculars (as opposed to short-term lingua francas) is in question. There are many examples of short-term ‘high status’ language introduction followed by eventual failure to establish the new language permanently amongst a large indigenous population – Alexander the Great, the Normans in England, the Mongols and the Ottomans all stand out. Even colonial European powers in countries such as Vietnam (French), Indonesia (Dutch), India and Malaysia (English) had little linguistic success in this regard. Latin was spread mainly through the army and its soldier-settlers, rather than because all of the native populations of the Roman Empire learnt it and abandoned their own languages. After the end of that Empire, the linguistic daughters of Imperial Latin continued for the next 1500 years as national vernaculars in less than half of the Empire's second century CE extent.

The only potential example of ‘elite dominance’ on the scale of a major potential language family known to me is that of the Iberians in the Americas, which did not involve large quantities of settler migration, unlike that of the later British who travelled to North America and Australasia as migrant families and not simply as a male-dominated and estate-owning elite. However, the Old World diseases introduced during the sixteenth century ensured that many American indigenous peoples and their languages died together, up to a staggering 90% of the population in some regions (Koch et al., 2018), paving the way for an eventual domination by the Spanish and Portuguese languages amongst the admixed European, African and indigenous populations of the colonial era (Ongaro et al., 2020). The adoptions of Spanish and Portuguese by indigenous populations in the Americas were not just simple cases of language shift imposed by an elite minority through landscapes of intact and willing communities. Where Native American communities survived the impact of disease, as in the Arctic and parts of Mesoamerica, Amazonia and the Andes, their indigenous languages survived, and still do so.

Successful and widespread language families, therefore, required substantial migrations by their original speakers in order to exist. Within the global records of archaeology and ancient DNA, the most substantial Holocene migrations were those of farming populations who had come to depend upon a portable repertoire of domesticated plants and animals, who were undergoing substantial population growth, and who expanded mainly amongst antecedent populations of hunter–gatherers. We can identify such migrations very clearly in the Neolithic expansions out of the Fertile Crescent, through Europe, North Africa and into the Eurasian steppes. I suspect these migrations carried the deepest foundations of the Indo-European and Afro-Asiatic language families, although, as I discuss in the next section, it might always be very difficult to prove this using linguistic evidence alone.

Similarly, Neolithic expansions out of the rice and millet agricultural homelands in central and northern China spread through much of eastern Asia and the Indo-Pacific region. The Transeurasian dispersal was one example, and I strongly suspect that the Sino-Tibetan, Austroasiatic and Austronesian language dispersals were others.

## ‘Complete’ vs. ‘truncated’ language families, and homeland questions

To what degree can linguists ever hope to reconstruct the homeland region of a language family with a level of certainty that everyone finds convincing? It is apparent that Austronesian, for example, is agreed by virtually all linguists to have had a proto-language that can be sourced to Taiwan (although Taiwan, of course, is only the furthest back one can trace, and certainly not an ultimate homeland). Likewise, there seems to be little disagreement over the location of the Bantu homeland in the vicinity of Cameroon.

Indo-European, on the other hand, has been given suggested homelands in remarkably diverse places during the past century or so. We might wonder why Austronesian should be so much clearer in terms of its homeland location than Indo-European. This situation seems to reflect a major problem in understanding the deep prehistories of many of the larger language families. Linguists and archaeologists generally operate from the assumption that the homeland of any given language family can be reconstructed from comparison of existing and historically recorded subgroups and their component languages. But can it, always?

Completeness of subgroup survival is no doubt a very good assumption for language families such as Austronesian and Bantu. These language families are ‘complete’ in the sense that there has been no large-scale linguistic replacement within their distributions, and their homelands and patterns of geographical unfolding are still fairly easy to read, even if there have been, here and there, localised regions of replacement. A good example of such replacement would be the process that Robert Blust terms ‘linguistic levelling’ in the Philippines (Blust, 2019), whereby some closely related Austronesian languages have apparently expanded, relatively recently, at the expense of many of their previously founded linguistic relatives. Such levelling, however, does not affect identification of a Proto-Austronesian homeland in Taiwan.

Other language families are far more recalcitrant. A major problem for historical linguists comes with those, like Indo-European, that have been ‘truncated’ by multiple layers of population expansion and language replacement, in many cases so long ago that no clear traces remain of exactly what happened. As Henry Hoenigswald stated many years ago with respect to Indo-European:

Hittite and Tokharian … are now extinct; there are other splinters, barely known to us, of which the same is true, and we may conjecture, though with meager profit, that there were many additional groups, now lost without a trace. (Hoenigswald, 1969)

My own opinion on Indo-European is that the language family as it exists today reflects more than one major episode of human migration, not all from one homeland. Relatively truncated language families like Indo-European survive today as a number of subgroups related in a rake-like rather than tree-like fashion, with each subgroup being usually quite clear-cut in terms of its internal membership, but difficult to relate to other subgroups in the family in any nested historical sequence of separation from a common core of ancestry. It does not take much linguistic understanding to realise that the language families in this category cause remarkable quantities of dispute about history and origins.

Examples? Apart from Indo-European they certainly include Afro-Asiatic, Austroasiatic, and many of the American families. The last express this problem mainly because of the huge swathes of linguistic extinction caused by the spreads of colonial languages, especially English, Spanish and Portuguese. Many of them only survive in fragmentary form. I doubt, using linguistic reasoning alone, that we will ever know the homeland locations for all of these relatively truncated language families to the satisfaction of all interested parties, even if some have been more truncated than others. That is why the multidisciplinary approach exemplified in this set of articles is so important.

I am also inclined to include Transeurasian within this group of relatively truncated language families. The Transeurasian languages as they survive today have suffered very extensive language replacement. Sinitic languages and Russian (neither, of course, Transeurasian) have wreaked havoc on its presumed former distribution, as also must have the expansions of the existing Transeurasian subgroups and single languages (especially Mongolian, Korean and Japanese).

This is apparent from the Bayesian ‘Densi Tree’ of the five major subgroups presented by Martine Robbeets and Remco Bouckaert (2018: 158), and from the quantity of past disagreement about the order of separation of the component subgroups from each other. Robbeets and Bouckaert do favour a specific order of separation derived from their statistical methodology, and this begins with a binary separation at the level of Proto-Transeurasian (c. 5000 BC) that led eventually to Koreanic and Japonic on one hand, and Tungusic, Mongolic and Turkic on the other. However, internal differentiation within the Japonic, Tungusic, Mongolic and Turkic subgroups (excluding Korean, which is a single language today) only dates from about 500 BC onwards. The previous millennia of Transeurasian linguistic evolution are relatively opaque in terms of population movements and separations. It is possible that some original subgroups created during the break-up of Proto-Transeurasian no longer exist, as Martine Robbeets acknowledges as a general principle in her article with Chuan-chou Wang on the Tungusic homeland:

original linguistic diversity may have been erased and it may no longer be possible to pinpoint the homeland using the diversity hotspot principle. (Wang & Robbeets, 2020: 3)

## The Transeurasian language family and the Farming/Language Dispersal Hypothesis

Seven papers in the set of nine deal directly with questions about the spread of the Transeurasian language family or its major subgroups, together with the human genomes and archaeological cultures that might have been attached to it. All touch on the Farming/Language Dispersal Hypothesis in one way or another.

### Transeurasian textile production

Sarah Nelson, Irina Zhushchikovskaya, Tao Li, Mark Hudson and Martine Robbeets (2020) discuss Transeurasian prehistory from the perspective of textile technology. The authors reiterate the idea previously put forward by Martine Robbeets (e.g. Robbeets, 2017) that the Transeurasian languages spread with millet agriculture, commencing soon after 5000 BC from a source in eastern Inner Mongolia and Liaoning, in the lower Liao drainage basin. Here existed an early millet-cultivating population that lived in sedentary farming villages, well attested in the archaeological record as the Xinglongwa culture, and its Zhaobaogou and Hongshan successors. The Bohai Sea separates this area from the larger region of early millet cultivation along the Yellow River to the south, and from both geographical and linguistic perspectives it makes good sense to see the Yellow River as the source of Sino-Tibetan languages (Sagart et al., 2019; Zhang et al., 2019), and the Liao as the source of the unrelated Transeurasian languages.

However, in terms of purely linguistic dating (using Bayesian analysis) of individual subgroup proto-languages, these authors calculate much younger dates, only around 2000 BC for Proto-Japano-Koreanic and Proto-Turko-Mongolic, and younger again for each individual Transeurasian subgroup, as noted previously. The initial millennia of Transeurasian expansion have therefore been overlain by the expansions of the subgroups that survive today. Unfortunately, we cannot understand with certainty the configuration of a prehistoric linguistic landscape that has been replaced, but Nelson and colleagues are able to point to a common terminology for textile production, associated with spindle whorls for spinning fibre, that can be reconstructed to Proto-Transeurasian.

This is therefore a valuable paper, based on research in both linguistics and archaeology, that reinforces through the presence of spindle whorls the existence of some kind of textile technology in the earliest farming cultures in northeastern Asia, including Xinglongwa in Manchuria, Zaisanovska in the Russian Far East (Primorye), Early Chulmun in Korea and Yayoi in Japan. Interestingly, older cultures with basically hunter–gatherer economies, such as Incipient Chulmun in Korea and Jomon in Japan, lack these artefacts.

A reconstructed ancestral vocabulary for spinning and weaving in the earliest Neolithic of Northeast Asia certainly illuminates our understanding of the cultural context for the inception of the Transeurasian language family, even if it does not pin down an exact region of origin. However, I do have one small comment. The authors refer to ‘loom weights’, the implication being that the people concerned knew the use of a vertically weighted loom, as opposed to the backstrap loom characteristic of many parts of Southeast Asia in the ethnographic record (and incidentally also represented in bronze in the Dian Bronze–Iron Age artefacts of Yunnan, late first millennium BC – Rawson, 1983: figs 13–16). Vertical looms were certainly present in northern China during historical times, at least from the Han Dynasty onwards (Zhao et al., 2017), but in this article the ‘loom weights’ illustrated for the Manchurian Neolithic are not convincing. One (fig. 3, no. 20) is almost certainly a net weight for fishing, similar to notched pebbles found in Neolithic archaeological sites in the northern Philippines and Taiwan (Bellwood & Dizon, 2013: fig. 8.9). We do not know what kind of loom was used in Manchuria around 5000 BC, but the backstrap variety, in terms of its Bronze Age existence and ethnographic distribution, would seem more likely than the vertical frame type.

### Korea

Jangsuk Kim and Jinho Park (2020) raise the question of whether Transeurasian languages initially spread into the Korean Peninsula with the millet-cultivating Chulmun Neolithic culture around 3500 BC, or with the rice-cultivating Mumun late Neolithic and Bronze Age culture about 1500 BC. Martine Robbeets (2017) has favoured a Chulmun genesis of Proto-Japano-Koreanic, with an arrival of the ancestral language in Korea c. 3500 BC, followed by a separation between the Koreanic and Japanic subgroups at about 2000 BC (as shown in Robbeets & Bouckaert, 2018: fig. 8). However, Kim and Park favour a much younger, Mumun, arrival of Japano-Koreanic languages because of the strong and sharp appearance of the Mumun culture, with rice, in the Korean archaeological record.

The archaeological evidence, however, seems a little too weak to me for any final decision about how Transeurasian languages first entered Korea, even if their presence there during the Mumun phase seems fairly evident, given the impending movement of a Japonic language from Korea onwards to Japan around 900 BC. New plant-genetic data support a movement of a temperate variety of *japonica* rice to Korea after 2000 BC (Gutaker et al., 2020), so a Mumun immigration into Korea with rice cultivation is likely. However, to disallow the preceding Chulmun Neolithic phase as the initial Transeurasian language context in Korea seems to me to be premature.

For instance, Archaeobotanist Gyoung-Ah Lee (2011) has noted the archaeobotanical presences of both foxtail and broomcorn millet in several Chulmun sites, and the previously discussed article by Nelson and colleagues suggests that weaving technology was introduced to Korea during Early Chulmun times. In this regard, Chulmun archaeology records at least two major cultural introductions to Korea, presumably from or via the Liao Valley.

I lack sufficient linguistic familiarity with this part of the world to adjudicate further between these two opinions, but the question of dating the initial arrival of Transeurasian languages in Korea still seems to be open, especially given the lack of archaeological data from North Korea. Indeed, this debate reminds me of something I have felt many times in connection with the Language/Farming Dispersal Hypothesis. To test the hypothesis at the level of a whole language family is an exercise very different from, and often more difficult than, that of trying to equate specific languages with specific prehistoric cultures. The whole can sometimes be greater than the sum of its parts.

### Japan

The first article on Japan, by Elisabeth de Boer, Melinda Yang, Aileen Kawagoe and Gina Barnes (De Boer et al., 2020), is truly multidisciplinary, covering linguistics, genetics and archaeology. The genetics section (by Melinda Yang) regards the Jomon early pottery-using people as indigenous to Japan since at least an uncertain molecular clock date for their separation from Asian mainland populations between 38,000 and 18,000 years ago, albeit with periodic contact since that time span with other East Asian mainland coastal populations. However, for Yang, the Jomon show no close genetic relationship with Southeast Asian pre-Neolithic Hoabinhians from Laos and Malaysia, and this is a contrary view to that published in other recent papers by Hirofumi Matsumura et al. (2019: craniofacial analysis) and Hugh McColl et al. (2018: genomics). Doubtless, biological anthropologists and geneticists will need to resolve these conflicts, and perhaps ancient DNA from pre-Neolithic people in central China will be needed to do so.

This archaeology section in this article reinforces the widespread view that Jomon people were mainly hunter–gatherers who practised minor cultivation until rice and millets were introduced from the Mumun culture in Korea at about 900 BC. This introduction of agriculture commenced the Yayoi culture of Japanese late prehistory, and was also the putative context for the introduction of the ancestral Japonic language(s) into Japan, from Korea. The arrival of the Yayoi into Jomon Japan was thus a specific case of farming/language dispersal.

Nowadays, there seems to be general agreement amongst Japanese linguists and archaeologists that the Yayoi culture witnessed a gradual spread of Japonic-speaking immigrants through Japan, from Kyushu to as far as northeastern Honshu, mixing all the way with the existing Jomon populations, who probably spoke languages related to modern Ainu. The final sections of this article examine this process of spread and mixing through the archaeological record, and through the question of language replacement, in the Tohoku Prefecture of northeastern Honshu.

The second paper on Japan, by Mark Hudson, Shigeki Nakagome and John Whitman (Hudson et al., 2020), discusses Jomon and Yayoi prehistory from the perspective of Kazuro Hanihara's (1991) dual structure hypothesis, an early claim for a Jomon to Yayoi succession in Japanese prehistory. They find that it still works well, and that the expansion of the Japonic languages (Japanese and Ryukyuan) out of Mumun Korea can be successfully modelled by the Farming/Language Dispersal Hypothesis, as foreshadowed in the previous article by De Boer et al. Japanese and Ryukyuan are believed to have separated during the Yayoi period and onwards, but they also point out that population movements from Korea into Japan continued well into the Kofun and Nara periods, to perhaps as recently as AD 800. Jomon populations in Japan were tenacious, surviving continuously alongside the speakers of Japonic, especially in the case of the present-day non-Transeurasian Ainu languages of Hokkaido.

### Tungusic

Two articles cover questions of Tungusic origins and ancestral genetics. Chuan-chao Wang and Martine Robbeets (2020) discuss the homeland of Proto-Tungusic, placing it in the Lake Khanka region of the lower Amur Valley in the Russian Far East. They regard the break-up of Proto-Tungusic as an Iron Age phenomenon, dating between 600 BC and AD 700, and associated with millet farming. The biological population itself, however, appears already to have been in the Amur region for at least 8,000 years, and this is established from ancient DNA analysis in an adjacent paper by Yinqiu Cui and many collaborators (Cui et al., 2020). They use ancient DNA from a Neolithic site in Heilongjiang to suggest that the Amur people of the Zaisanovska archaeological culture around Lake Khanka (southern Primorye) were ancestral to modern Tungusic speakers, and that millet farming spread into Primorye from Hongshan cultural sources (3500–3000 BCE) in the West Liao Valley.

Both articles therefore suggest a population continuity in the Amur River basin since at least 8,000 years ago, but also agree on a spread of millet farming with Transeurasian languages and presumably their speakers from the West Liao region. Yet another newly published analysis with Yinqiu Cui as a collaborator, of ancient genomes from the Yellow, Liao and Amur valleys, places Hongshan individuals close to Amur Neolithic and Iron Age individuals in a principal components plot (Ning et al., 2020: fig. 2a), closer than to other Neolithic individuals from the Yellow River. Perhaps the Hongshan culture, that represented a peak in terms of population size in the Liao Neolithic, was a period of significant migration that carried Transeurasian languages far northeastward into the Amur Basin.

### Turkic

The article by Junzo Uchiyama, Christopher Gillam, Alexander Savelyev and Ning Chao (2020) discusses population dynamics in northern Eurasian forests. It has two rather separate parts. The first discusses the *Homo sapiens* settlement of northeastern Asia by Upper Palaeolithic populations, who reached 50°N by about 45,000 years ago. During the Last Glacial Maximum, at about 20,000 years ago, some of these populations retreated to warmer regions such as the Amur Valley and Palaeo-Honshu, where postglacial pottery traditions developed that eventually spread westwards across northern Eurasia to reach the Ertebølle Mesolithic culture in Scandinavia.

For me, the most exciting suggestion in this section is that the Upper Palaeolithic populations that settled the American continents from northeastern Asia around 15,000 years ago, via Beringia and Alaska, could have originated in Japan or Sakhalin. The bifacial points and microblades that occur in Japan at this time fit this picture remarkably well (Davis et al., 2019: fig. 5; Tanomata & Tabarev, 2020), and these authors reject the idea that the first Americans underwent a long period of standstill in Beringia. If there was a standstill, it is more likely to have occurred in maritime northeastern Asia, in a region that included Japan.

The later section in this article deals with Turkic expansion during the first millennium BC from a possible forest/steppe boundary homeland in eastern Mongolia. The Proto-Turkic vocabulary had terms for both domesticated crops (broomcorn millet, wheat and barley) and animals (horse, cattle and sheep). One might wonder why this section on Turkic is included in an article that is otherwise mostly about the Palaeolithic, but the reason seems to be that Proto-Turkic is claimed to have had connections with the northern Eurasian forests, like the Japanese Upper Palaeolithic. Clearly, it had an agricultural and pastoralist basis that renders it as a potential example of the Farming/Language Dispersal Hypothesis. As such, it demonstrates that the hypothesis need not always relate to the very first farmers to inhabit any particular geographical situation – food producing populations can migrate at any time if they have a growing population and little opposition.

There are two more articles in this set of articles, neither intersecting with the Farming/Language Dispersal Hypothesis. Alexander Savelyev and Choongwon Jeong discuss the identities of the migratory Xiongnu, Huns, Rourans and Avars who appear in the early annals of central Asian history. Gyaneshwer Chaubey and George van Driem discuss how speakers within different language families in East Asia carry specific haplotypes within the Y chromosome macro-haplogroup O, and then present East Asian prehistory as a reflection of the migrations of these male-borne haplogroups. Needless to say, their results do not overlap with those of the other authors who discuss Transeurasian origins and dispersals in this set of articles.

How to sum up? The original Jena conference was organised by Martine Robbeets as a platform for specialists from linguistics, genetics and archaeology to come together and write joint assessments of specific topics in Transeurasian prehistory. However, seamless and tightly focused discussions drawn from completely separate disciplines can sometimes be difficult to create, and some of the articles reviewed here certainly illustrate this difficulty. This does not make them any less readable on a section-by-section basis, but it does mean that readers need to sometimes stop and think about how to put the whole of a presentation together.

Has progress been made in understanding Transeurasian prehistory? The answer for me is certainly yes, especially for people who are not specialists in the field. I see some very positive evidence that the original speakers of Transeurasian languages were millet farmers in northeastern China about 7,000 years ago, and that they began to spread soon after this date around the northern periphery of the area to their south that was already being occupied by early Sino-Tibetan language speakers. The original Transeurasian subgrouping structure that must have developed from these initial Neolithic dispersals was later obscured by the expansions of the major subgroups that exist today. In turn, the distributions of these major existing subgroups have been cut back by the subsequent expansions of Chinese and Russian, and by state-level languages such as Mongolian, Korean and Japanese within the Transeurasian subgroups themselves.

I know that there are many scholars of prehistory today who regard attempts to read the past on a broad scale, using multidisciplinary data and hypotheses, as misleading, because they tend to impose a global view over the top of a vast series of independent non-global observations that might not always agree. However, neglect of broad hypotheses can be just as misleading, because then the forest can vanish so far behind the trees so that we see nothing global at all. I see the future for investigations into human prehistory as combining both approaches. There is nothing to be gained by over-specialisation, and we must never neglect the importance of stating what we believe to be the most likely hypothesis to explain any given situation in terms of the data that are currently available. This is where the Farming/Language Dispersal Hypothesis comes in.

Martine Robbeets and her colleagues are to be congratulated on putting together such a genuinely multidisciplinary collection of viewpoints on one of the world's most interesting archaeolinguistic arenas.
